# Occupation-Based Practice Among Iranian Occupational Therapists: A Cross-Sectional Survey

**DOI:** 10.1155/oti/8250183

**Published:** 2025-05-09

**Authors:** Esmail Sadeghi, Mahnoosh Khanipour, Mohammad Khayatzadeh-Mahani, Jamileh Abolghasemi, Afsoon Hassani Mehraban

**Affiliations:** ^1^School of Rehabilitation Sciences, Iran University of Medical Sciences, Tehran, Iran; ^2^Occupational Therapy Department, Rehabilitation Research Center, School of Rehabilitation Sciences, Ahvaz Jundishapur University of Medical Sciences, Ahvaz, Iran; ^3^Biostatistics Department, School of Public Health, Iran University of Medical Sciences, Tehran, Iran; ^4^Occupational Therapy Department, School of Rehabilitation Sciences, Iran University of Medical Sciences, Tehran, Iran

**Keywords:** attitude, clinical experience, environmental barrier, knowledge

## Abstract

**Introduction:** Despite the global focus on occupation-based practice (OBP), limited research exists on its implementation in Iran. There is a lack of evidence on how OBP is practiced and the factors affecting its use among Iranian occupational therapists, which is essential for addressing barriers and promoting a more occupation-centered approach in clinical settings.

**Objectives:** This study is aimed at determining the knowledge, attitudes, interests, usage rates, and environmental factors affecting OBP among Iranian occupational therapists and examining the relationship between sociodemographic factors and OBP.

**Study Type:** This work is a cross-sectional analytical study.

**Data Collection Location:** The data collection location of this study is the private and government clinics across Iran.

**Methods:** A total of 202 participants completed demographic and OBP questionnaires. Key aspects such as total performance, usage rate, attitudes, knowledge, interest, and environmental factors were evaluated. Data analysis was conducted using SPSS software, employing statistical tests including ANOVA, Pearson's correlation, and *T*-test.

**Results:** The total OBP performance among Iranian occupational therapists is 64%, and 60.7% of them use OBP approaches. They have a positive attitude (83.6%) and high interest (78.3%) towards these approaches. However, environmental factors (44.7%) and an average level of knowledge (65.6%) have hindered the full utilization of these approaches. The average OBP score among occupational therapists in government settings was significantly higher than in private centers (*p* value < 0.001). Additionally, occupational therapists with a PhD scored higher than those with a bachelor's or master's degree (*p* value < 0.001). The level of work experience has a significant correlation with the total OBP score (*p* value < 0.05).

**Conclusion:** This study examines the factors affecting OBP adoption in Iran, showing that while therapists have positive attitudes and interest, environmental barriers and limited knowledge hinder full implementation. Government-employed therapists and those with PhDs engage more, and work experience is crucial. The findings stress the need for targeted education and systemic changes, contributing to global insights on OBP integration.

## 1. Introduction

The use of occupation as a primary therapeutic tool and modality has been prominent in the paradigm of occupational therapy (OT) from the very beginning, and the pioneers of this field introduced occupation as the core of the OT profession. Since the early 20th century, occupational therapists have used occupation as an initial intervention for individuals with mental illness and physical disabilities. The development of this profession was influenced by moral treatment, which was common in the 18th and 19th centuries as a treatment for mental patients. The assumption of moral therapy was that engaging in various daily occupations could restore an individual's health and functioning [1]. The latest version of the Occupational Therapy Practice Framework (OTPF-4^1^) defines occupation as the personal daily activities that individuals perform as members of a family and community to pass the time and bring meaning and purpose to life. Occupation includes things that people have to do, want to do, and are expected to do. Occupations can be completed by performing several activities and can lead to various outcomes. Occupations are classified into a wide range of daily life activities, instrumental activities of daily living, health management, rest and sleep, education, work, play, leisure, and social participation [2]. Occupation-based practice (OBP) are defined as activities that support performance in the areas of ADL, IADL activities, rest and sleep, education, work, play, leisure, and social participation [3]. Key features of OBP include based on the theory of occupation, interaction with the client's context, use of occupational language in discourse and professional documentation, and the use of occupation throughout the OT process. OBP leads to the formation of a strong professional identity in therapists and clarity of role in the field of OT [2].

Different authors have defined OBP as the clinical use of occupation-based intervention (OBI) [4]. OBI focuses on the use of meaningful activities or occupations to enhance health, well-being, and participation in daily life. OBI includes identifying the goals and priorities of the client and creating treatment plans that involve activities meaningful and relevant to the client's life. OBI can be used for clients of any age and with a wide range of conditions, including physical disabilities, mental health issues, and developmental disorders. The goal of OBI is to help clients develop the skills and abilities they need to participate in meaningful activities and achieve their goals [5].

The review of texts considers the use of OBP to be useful. Occupation-based OT improves the general health of stroke patients, emotional roles [6], and occupational performance in activities of daily living [3]. Studies have also shown that participation in meaningful activities, through stimulating the nervous system, improves individuals' health and well-being and reduces stress caused by illness [7]. Additionally, a meta-analysis showed that occupation-based cognitive rehabilitation is beneficial for improving daily functioning and psychosocial performance in patients with brain injuries [8]. Additionally, OBIs in various clinical settings, including hand therapy, neurological rehabilitation, and burn injury rehabilitation, have reported favorable outcomes [9]. OBIs are set in real-life living environments [10] and enhance interaction and motivation to improve occupational performance [11, 12] and functional independence [13], IADL [14], and social participation [15] for stroke patients. Additionally, their effectiveness in improving upper limb function in stroke patients has been proven [3, 16–20]. Additionally, OBIs play a role in improving participation and occupational performance in the rehabilitation of addicts [21] and enhancing participation in children with physical disabilities [22].

Various studies have been conducted to determine the extent of occupational therapists' use of OBP interventions. A study conducted in 2019 on members of the American Occupational Therapy Association showed that occupational therapists spend 34% of their time on OBP [4]. This percentage among American occupational therapists in the hand domain was 21%–30% in 2010 [23] and in 2022, most participants (57.7%) implemented OBI at least 75% of the time [24]. However, a study conducted in 2023 found that 41% of respondents reported using OB activities with their clients 26%–50% of the time [25].

However, the reported use of OBP by occupational therapists does not always match the actual use of these interventions. In a 2014 study among occupational therapists in New Hampshire, United States, 73% reported that they always or often used OBP. However, when examining the performance of their evaluation, only 22% of the stated evaluation methods reflected this perspective [9]. In 2022, 124 occupational therapists working in hospitals and rehabilitation institutions in Norway responded that they use OBP assessment and intervention methods in 76% of their evaluations and 84% of their daily or weekly interventions. However, upon reviewing the stated assessment methods, it was found that only 26% of the mentioned assessment methods were OBP, and only 38% of the intervention methods stated by the occupational therapists were OBP [26].

OT in Iran has evolved significantly since its establishment, with 15 OT schools and approximately 4000 registered occupational therapists serving a population of 85 million people. Despite this growth, the integration of OBP remains limited due to various systemic and professional challenges. Several barriers and limitations exist for the implementation of OBP in Iran, including inadequate educational programs such as the lack of courses related to OBP, the mismatch between theory and practice, and inexperienced professors; lack of public and professional awareness of the OT profession and its services; unsuitable clinical environments such as the mismatch between clinic environments and OBP and the lack of tools and equipment; cultural and social challenges such as cultural differences and the low educational and economic levels of clients; insufficient research on the effectiveness of OBP; and time pressure and financial challenges for therapists and clients [27]. These barriers highlight the need for research exploring the current state of OBP in Iran and identifying factors that influence its adoption.

Studies in Malaysia [5, 28, 29], the United States [4, 23, 24], Australia [30], and Norway [26] have examined factors affecting OBP, such as organizational policies, limitations in therapists' knowledge and skills, lack of time, and limited resources. While these findings provide a foundation, the context of Iran presents unique challenges, including a lack of public and professional awareness of the OT profession and its services, cultural and social challenges such as cultural differences and the low educational and economic levels of clients, insufficient research on the effectiveness of OBP, and financial challenges for both therapists and clients. Understanding how these global findings apply to Iran is essential for developing strategies that are suited to local conditions.

Research has shown that OBP is a valuable approach among occupational therapists. However, some occupational therapists prefer to use a medical approach focused on body functions instead of the OBP approach [26], and there is also a notable discrepancy between the description of OBP and its implementation in interventions [31]. On the other hand, Rudman and Dennhardt demonstrated that cultural factors, the economy, the labor market, and the media play a significant role in shaping individuals' understanding of occupational identity. The authors argue that occupational identity is not solely determined by individuals' personal experiences but is also influenced by cultural beliefs and values. Furthermore, cultural norms and expectations influence the types of occupations, and cultural diversity can significantly impact the concept of occupational identity. Individuals from different cultural backgrounds may have varying expectations and priorities regarding their jobs, which can affect their sense of identity and satisfaction in their chosen profession. Therefore, when examining an individual's understanding of their occupational identity, paying attention to the cultural context is important [32]. Iranian culture, due to the diversity of ethnic groups with unique values and beliefs, is distinct and different from the cultures of other countries [33]. The context of the physical environment (natural environment, streets, buildings, and equipment) in Iran differs from Western countries where OBP has developed, as well as the social contexts in terms of norms; role expectations; social routines; and organizational, political, and economic systems. Cultural context has a profound impact on occupation and OBP, and it seems that implementing OBP in developing countries like Iran is more challenging than in developed countries due to differences in cultural, physical, and social contexts [27].

Since OBP leads to the formation of a strong professional identity in therapists and role clarity in the field of OT [34], on the other hand, the cultural context has a profound impact on occupation and OBP. Considering the contextual differences between Iran and developed societies, it seems that Iranian occupational therapists and clients have different experiences and perspectives regarding the performance of OBP. Consequently, it is necessary to clarify the views, attitudes, knowledge, and awareness of Iranian occupational therapists regarding the concept of occupation and OBP, the importance they attribute to it and consider it effective, the extent to which Iranian occupational therapists use OBP in clinical work, and the barriers and facilitators in implementing OBP.

## 2. Objectives

This study is aimed at assessing the extent of OBP implementation among Iranian occupational therapists and identify key factors influencing its adoption. By analyzing therapists' perspectives through a questionnaire survey, the study provides an overview of existing conditions and highlights potential barriers and facilitators. The findings may inform future research and professional initiatives aimed at enhancing OBP integration in Iran.

## 3. Methods

### 3.1. Participants

In this cross-sectional study, targeted licensed occupational therapists practicing in Iran were included. Inclusion criteria were (1) holding a valid OT license from the Iranian Ministry of Health and (2) working in either government or private healthcare settings. According to national records, there are approximately 4000 registered occupational therapists in Iran. The sample size with a confidence level of 95% (1-*α*) and a test power of 80% (1-*β*) was calculated based on a correlation coefficient (*r*) of 0.2 to estimate the largest possible sample size, which was estimated to be 194 individuals. Assuming a response rate of 40%, 485 individuals were considered for the distribution of the questionnaire. Random numbers were generated using SPSS software (Version 27), and individuals were selected randomly. An email containing the informed consent form for participation in the research, the demographic questionnaire, and the Occupation-Based Practice Questionnaire was sent to them. In the end, 202 people responded to the emails. This study was conducted from August 2023 to February 2024.

 $$\begin{array}{c} n=\frac{{\left(Z_{1-\alpha /2}+Z_{1-\beta }\right)}^{2}}{{\left(1/2\;\ln\left(1+r/1-r\right)\right)}^{2}}+3=\frac{{\left(1.96+0.84\right)}^{2}}{{\left(1/2\;\ln\left(1+0.2/1-0.2\right)\right)}^{2}}+3\sim 194. \end{array}$$

### 3.2. Data Collection Tools

In this research, the Occupation-Based Practice Questionnaire and the demographic questionnaire will be used. The Occupation-Based Practice Questionnaire was designed by Khayatzadeh Mahani et al. in 2022 to evaluate the knowledge, attitude, and implementation of OBP and the factors influencing it among Iranian occupational therapists working in OT clinics and to examine the current status of OT in Iran regarding the implementation of OBP. This questionnaire consists of 37 items, with Items 1–11 titled “Level of Use of Occupation-Based Practice,” Items 12–21 titled “Environmental Factors Affecting the Implementation of Occupation-Based Practice,” Items 22–30 titled “Occupational Therapist's Attitude Towards Occupation-Based Practice,” Items 31–34 titled “Occupational Therapist's Knowledge About Occupation-Based Practice,” and Items 35–37 titled “Occupational Therapist's Interest in Occupation-Based Practice.” Participants rated their clinical performance on a scale of five levels (*Completely*: full information, constant use, and full agreement with the statement in question; *Mostly*: a lot of information, frequent use, and agreement with the statement in question; *Moderate*: some information, occasional use, and no specific opinion regarding the statement in question; *Slightly*: incomplete information, little use, and disagreement with the statement in question; and *None*: no information, no use, and complete disagreement with the statement in question). The questionnaire was adapted from previously validated instruments measuring OBP implementation and barriers. To ensure validity, expert occupational therapists reviewed the content for relevance to the Iranian context. Face and content validity were evaluated by an expert panel of 21 occupational therapists, each with at least 10 years of experience in the field. Construct validity was examined via exploratory factor analysis (EFA) with factor loadings above 0.40 considered acceptable. Reliability was assessed using Cronbach's alpha, with an overall reliability coefficient of *α* = 0.94 indicating strong internal consistency. The ICC was used to determine test–retest reliability. The calculated ICC value for each item ranged from 0.719 to 0.948, showing good to excellent reliability [35].

### 3.3. Measures

#### 3.3.1. Outcome Variables

This study examined total OBP performance, usage rate, knowledge, attitude, interest, and environmental factors measured using a validated Occupation-Based Practice Questionnaire.

#### 3.3.2. Independent Variables

Demographic characteristics were treated as independent variables and included gender (male and female), educational degree (bachelor, master, and PhD), clinical experience (years), field of work (mental health, physical health, and psychiatry), and place of service provision (private center and government center). Participants provided this information by completing a researcher-designed demographic questionnaire specifically developed to capture relevant demographic data.

### 3.4. Statistical Methods

For data analysis, SPSS software (Version 27) was used. The normality of the data distribution was examined and confirmed using the Kolmogorov–Smirnov test (*p* > 0.05). Additionally, to evaluate the homogeneity of variance of the performance score based on occupation in different groups, Levene's test was conducted (*p* > 0.05). For the analysis of the general characteristics of the participants, descriptive statistics including frequency, percentage, mean, and standard deviation (SD) were used. To compare the mean performance score based on occupation between the two genders, the independent *T*-test was used, and to compare the mean in the variables of place of service provision and field of work, the one-way ANOVA was employed. In order to examine the correlation between work experience and the total performance score based on occupation, the Pearson test was used. The significance level for all analyses was considered to be *p* < 0.05.

### 3.5. Bias

In this study, there are several potential sources of bias:

Self-reporting bias: This study relies on self-reported data from questionnaires. This method is prone to social desirability bias, where participants may provide responses that they think are more socially acceptable or portray themselves in a better light. They may overestimate their use of OBP or their positive attitudes towards it.

Cross-sectional limitation: As a cross-sectional study, it only provides a snapshot at a specific time and cannot determine the causal relationship between factors and the implementation of OBP.

## 4. Results

### 4.1. Demographic Characteristics

Table 1 shows the demographic characteristics of the participants. Participants completed a questionnaire designed by the researcher to provide demographic information. The average work experience of the occupational therapists was 7.8 (SD = 7.12) years.

The mean, SD, minimum (Min), and maximum (Max) scores related to the usage rate, environmental factors, attitudes, knowledge, and interest of therapists are shown in Table 2.

**Figure 1:**
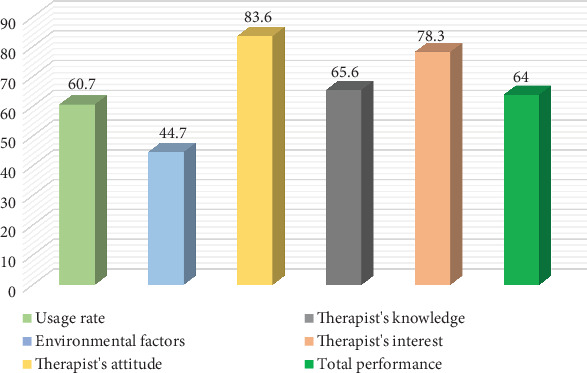
Percentage of usage rate, environmental factors, attitude, knowledge, interest, and total occupation-based performance among Iranian occupational therapists (*n* = 202).

Figure 1 shows that the total OBP performance among Iranian occupational therapists is 64%, and 60.7% of them use OBP approaches, indicating the implementation of these approaches in Iran. Iranian occupational therapists have a positive attitude (83.6%) and high interest (78.3%) towards these approaches. However, environmental factors (44.7%) and an average level of knowledge (65.6%) hinder the full utilization of these approaches.

The mean, SD, Min, and Max scores and percentage of environmental factors influencing OBP performance of therapists are shown in Table 3. The results of the environmental factors influencing the implementation of OBP among Iranian occupational therapists reveal a range of influences across different domains. The highest mean score was for motivation from clients and families (mean = 2.3, SD = 0.94), contributing 57.5% to the total, indicating that therapists are most encouraged by their clients and families to implement OBP. Administrator cooperation (mean = 2.0, SD = 1.05) also played a significant role, contributing 50% to OBP implementation. This highlights the importance of support from workplace management in facilitating OBP. On the other hand, access to occupation-based assessment tools (mean = 1.5, SD = 1.0) and compatibility of the community's physical environment (mean = 1.3, SD = 0.97) were among the least influential factors, contributing only 37.5% and 32.5%, respectively. The overall environmental factors score of 44.7% indicates that while there is a moderate level of support for OBP, there is room for improvement, particularly in creating more conducive physical environments and ensuring greater access to necessary assessment tools.

Table 4 presents the mean, SD, Min, and Max total performance scores based on occupation for Iranian occupational therapists, categorized by gender, educational degree, work field, and place of service provision. The results indicate that the place of service provision has a significant correlation with the OBP score, such that the average score in government centers is significantly higher than in private centers (*p* value < 0.001). Additionally, educational qualifications have a significant impact on the OBP score; occupational therapists with a PhD obtained higher scores compared to those with a bachelor's or master's degree (*p* value < 0.001), but no significant difference was observed between bachelor's and master's degrees (*p* value > 0.05). In contrast, the comparison of the mean occupational performance score between the two genders and in the work fields did not show a significant difference (*p* value > 0.05).

The correlation analysis between the amount of work experience and the OBP score shows that the number of years of work experience has a significant correlation with the total OBP score (*r* = 0.274, *p* value < 0.001, *N* = 202) and its various dimensions, including the use of OBP approaches, attitudes, knowledge, and therapists' interest (*p* value < 0.05). But no significant correlation was found between the level of work experience and environmental factors (*p* value > 0.05). These findings indicate that occupational therapists with more work experience have a positive attitude, specialized knowledge, and greater interest and usage of these approaches, highlighting the importance of experience in improving performance based on occupation.

## 5. Discussion

The findings of this study provide valuable insights into the implementation of OBP among Iranian occupational therapists while reflecting broader trends observed in international research. The results reveal a complex landscape of OBP implementation characterized by very positive attitudes but moderate practical use, influenced by various factors including education, experience, and environmental support. This study includes a diverse sample of 202 participants with different educational backgrounds, work experiences, and work environments.

Demographic data of the workforce in OT in Iran predominantly shows women, with the majority holding a bachelor's degree. This demographic profile may affect the implementation and overall understanding of OBP in Iran.

The discrepancy between high attitudes/interests and moderate knowledge/usage of OBP can be attributed to multiple factors. First, while occupational therapists in Iran recognize the value of OBP, their formal education may not provide sufficient hands-on training in occupation-centered interventions. Many OT programs still emphasize a biomedical approach, limiting exposure to occupation-based models. Second, environmental and institutional constraints—such as high caseloads, lack of OBP-specific resources, and restrictive policies—create barriers to translating interest into practice. Third, continuing professional development opportunities focusing on OBP remain limited, restricting therapists from advancing their theoretical understanding into real-world applications.

Despite positive attitudes towards OBP, implementation remains limited due to systemic and structural barriers. High patient caseloads force therapists to prioritize task-oriented interventions over time-intensive OBP methods. Institutional policies in many settings favor medical-model interventions, limiting flexibility for occupation-based approaches. Additionally, reimbursement structures often do not support occupation-centered interventions, discouraging therapists from integrating OBP into daily practice. These challenges suggest that while therapists are motivated to implement OBP, external constraints significantly hinder its widespread adoption.

While there is a high level of positive attitude, knowledge, and interest in OBP among Iranian occupational therapists, overall, 64% of Iranian occupational therapists reported high levels of OBP implementation, knowledge, attitude, and interest. This positive acceptance is consistent with findings from other countries with similar contexts to Iran, such as Pakistan. In Pakistan, 88% of occupational therapists recognized OBI as the core philosophy of their profession. It was also reported that 53.3% of therapists had an average of 45 min of clinical practice sessions focused on OBI, and 64.7% considered themselves well trained in this approach. However, 69.3% indicated that their professional practice in poststroke rehabilitation primarily followed the biomedical model, while 39.3% lacked a clear understanding of the occupation-based concept [36]. In Norway, where 72% of occupational therapists strongly believed that OBP is important [26], and the United States, where 79% believed that these measures are important for services provided in the treatment environment for hand injuries [37]. In New Hampshire, United States, the overwhelming majority of participants agreed that their interventions should directly address the occupational needs of patients [9]. The findings of the study on the implementation of OBP present a complex picture. The rate of OBP implementation reported by Iranian occupational therapists is average, with only 60.7% reporting frequent use. This gap between theoretical knowledge and practical application is an important finding that points to potential barriers in the implementation of OBP in Iranian healthcare settings. This difference indicates that while occupational therapists understand the value of OBP, they face challenges in translating this understanding into practice. These results align with the time allocated to OBP in various studies; for instance, in Pakistan, 72% of therapists use OBI in their practice for stroke patients [36]. In the study by Mulligan et al., 73% reported that they always or often used OBIs. However, when examining their assessment performance, only 22% of the stated assessment methods reflected this view [9]. In the study by Aas and Bonsaksen, occupational therapists reported that they use OBP assessment methods and interventions in 76% of evaluations and 84% of their daily or weekly interventions. Upon reviewing the stated assessment methods, it was found that only 26% of the mentioned assessment methods were OBP [26]. In the study by Lloyd et al. on members of the American Occupational Therapy Association, 34% of time was spent on these interventions [4]. In the study by Colaianni and Provident, 21%–30% of American hand therapists used these interventions [23], and in the study by Valdes et al., 41% of hand therapists reported that they dedicated 26%–50% of their time to occupation-based activities with clients [25].

The gap between occupational therapists' positive attitudes and the actual implementation of OBP observed in this study aligns with the findings of Di Tomasso et al. in Australia, where participants believed in the importance of OBP but did not necessarily use it as an intervention tool [30]. This points to a common challenge in translating the principles of OBP into practice across different cultural contexts. This is also in line with the findings of Valdes et al., who reported that while occupational therapists in the United States believed that addressing clients' occupational needs is important [25], the integration of occupation-based assessments with physical performance evaluations is not commonly practiced.

As a result, while Iranian occupational therapists demonstrate positive attitudes, good knowledge, and strong interest in OBP, environmental factors and practical implementation remain significant challenges. These findings align with global trends in the implementation of OBP and highlight the need for targeted interventions to bridge the gap between theory and practice in OT. Future efforts should focus on addressing environmental barriers, improving educational programs, and providing ongoing support for occupational therapists to effectively implement OBP in various clinical settings.

Environmental factors (physical fit of the community, positive feedback from clients and healthcare staff, cooperation from managers, clinical training, alignment of university courses, workplace facilities, access to assessment tools, and sufficient time for implementing OBP) emerged as significant barriers to the implementation of OBP, and 44.7% of occupational therapists reported that there was little or no environmental support. This finding is consistent with previous research on OBP facilitators among Iranian occupational therapists, who have deemed contextual factors essential for successful implementation [27]. The current study emphasizes the need to address these environmental challenges to increase the use of OBP. These findings suggest that while factors like motivation from clients and administrative support are strong enablers of OBP, the physical environment and access to assessment tools may present challenges in the implementation of OBP. In Jordan, occupational therapists face challenges in using assessment tools and OBIs. The reported challenges include a lack of equipment and resources, lack of cooperation from other medical team members, and insufficient privacy for patients [38]. In Pakistan, the implementation of OBIs in medical settings faces challenges, including the fact that only a few occupational therapists are trained and practice it, and the culture and healthcare system do not support the values of OBP. 36.7% of therapists believed that this method is not relevant in the cultural context of Pakistan. 41.3% reported that the duration of therapy sessions is limited [36]. In Malaysia, there are several barriers to the implementation of OBIs by occupational therapists, including (a) reliance on predetermined treatment protocols that primarily focus on the clinical symptoms of clients rather than the whole person, environmental factors, or relevant occupational performance; (b) insurance reimbursement policies; (c) limited resources such as space, materials, and tools; (d) insufficient time to provide services that require clients to have adequate knowledge to identify appropriate activities or to conduct OB assessments and interventions; and (e) the complexity of measuring occupational performance versus measuring body functions or performance skills [9].

Analysis of various studies on the implementation of OBIs (OBP and OBI) in OT shows that these interventions face different challenges and facilitators in different countries. In Iran, facilitating factors include appropriate educational programs and the positive attitude of therapists towards OBP, while challenges are related to lack of awareness and the clinical environment. In Malaysia, the implementation challenges of OBI include the lack of client motivation, limited knowledge and skills of therapists, and logistical and cultural issues [5, 28, 29]. In the United States [4, 23, 24] and Australia [30], similar barriers such as lack of time, limited resources, and pressure for measurable outcomes exist, while facilitators include peer support and a client-centered philosophy [4, 23, 24, 30]. In Norway, the barriers include lack of time and equipment. In general, therapists' experiences show that OBP and OBI are recognized as effective therapeutic tools, but their success depends on environmental, educational, and available resource factors [26].

This study found that occupational therapists with a PhD performed better in implementing OBP compared to those with a bachelor's or master's degree, suggesting that advanced education may improve the ability to effectively apply OBP. However, no significant difference was observed between those with a bachelor's degree and those with a master's degree. This may be due to several factors. First, the OT curricula at both the bachelor's and master's levels in Iran might not differ substantially in terms of OBP-focused training, with both levels prioritizing fundamental clinical skills over occupation-based methods. Additionally, many master's programs tend to emphasize specialization rather than strengthening OBIs, which could explain the lack of significant differences between these two groups. This result is consistent with similar studies in other countries, including the research by Colaianni and Provident in the United States, which emphasized the importance of advanced education in enhancing OBP implementation [23].

However, in the study by Aas and Bonsaksen, the results showed that a higher level of education does not have a significant impact on OBP implementation [26]. The inconsistency of these results may be related to various reasons, such as the smaller sample size in the Aas and Bonsaksen study. Additionally, in the Aas and Bonsaksen study, only a general comparison between the undergraduate level and higher education was made, with no distinction between master's and doctoral degrees. In contrast, the present study showed that occupational therapists with a doctoral degree had higher scores than both the bachelor's and master's degree groups, while no significant difference was observed between the bachelor's and master's degree levels.

The results of this study showed that the place of service provision has a significant impact on the OBP score. Government environments usually perform better than private centers. This may be due to better access to educational resources and equipment, which enables occupational therapists to develop their clinical skills more effectively and implement OBIs with higher quality. Additionally, in government settings, there is stronger supervisory support, which can reduce issues related to implementation quality and help occupational therapists work in a structured and supported environment. The presence of educational environments in government centers is also another influential factor, as these environments typically host OT students under the supervision of academic instructors. These conditions provide more opportunities for experiential learning and professional supervision, which contribute to the improvement of OBP. These findings are consistent with previous studies that have shown that government educational environments typically benefit from better access to resources, greater expertise, and stronger professional supervision, all of which contribute to improving the quality of OBP implementation [26].

This study showed that increased work experience is correlated with an improvement in the total mean score of OBP as well as with enhanced scores related to the use of approaches, positive attitudes, specialized knowledge, and interest in these approaches. The existence of this significant relationship between the level of clinical experience and OBP scores is consistent with some previous findings. For example, the study by Ahmad et al. in 2021 in Malaysia showed that occupational therapists with less than 5 years of experience perceive more barriers to implementing OBP compared to those with higher work experience. Additionally, younger occupational therapists identified more barriers to implementing these interventions compared to their senior counterparts [29].

However, the study by Aas and Bonsaksen showed that the level of clinical experience does not affect the implementation of OBP [26]. This discrepancy may be related to various factors, including the smaller sample size in the Aas and Bonsaksen study, which could have limited the statistical power of the findings. Additionally, this study mentioned that in Norway, factors such as lack of time, space, and equipment have been reported as the main barriers to the implementation of OBP. Especially, the lack of time and equipment has been associated with a decrease in the level of OBP implementation (according to self-reports by therapists). These environmental limitations may even restrict the ability of occupational therapists with high clinical experience to implement OBP, resulting in different outcomes compared to other studies.

This study showed that the field of work does not have a significant impact on OBP scores. This result indicates that OBP principles may be equally applicable in various OT fields. The lack of a significant relationship between the field of work and OBP scores contradicts some previous findings. For example, a 2021 study by Ahmad et al. showed that occupational therapists working in psychiatric and mental health settings reported more barriers to implementing OBI in Malaysia [29]. This discrepancy necessitates further investigation into the potential impact of the work environment on the implementation of OBP in different cultural contexts.

For future research, it is suggested that studies be conducted to compare reported and actual use of interventions, examine environmental barriers through qualitative studies, and test strategies to overcome these barriers through intervention studies.

## 6. Conclusion

The findings highlight that while Iranian occupational therapists generally demonstrate positive attitudes and high interest in OBP, its implementation is hindered by environmental constraints and moderate knowledge levels. A significant proportion actively use OBP, with government-employed therapists and those holding higher academic qualifications achieving better performance scores. Additionally, work experience positively correlates with OBP utilization, indicating its role in fostering proficiency. These results underscore the need for targeted interventions, such as enhanced educational programs and supportive workplace policies, to address knowledge gaps and environmental barriers, thereby promoting the effective implementation of OBP in diverse healthcare settings across Iran.

To improve OBP adoption, it is essential to enhance workplace support by offering OBP training for administrators and integrating OBP-focused coursework into university OT programs. Increasing client and family engagement through educational workshops and client-centered goal setting can further facilitate OBP use. Improving access to OBP-specific assessment tools and advocating for environmental modifications to make healthcare settings more OBP-friendly will also help overcome existing barriers. Furthermore, continuous professional development workshops will ensure therapists have the necessary skills to implement OBP effectively.

Future research should focus on qualitative studies exploring therapists' perspectives on OBP barriers and facilitators, as well as intervention studies to assess the impact of training programs and environmental changes. Comparative and longitudinal studies examining regional differences and tracking changes over time will provide insights into the effectiveness of these interventions. These efforts will help shape policies and practices that enable more widespread adoption of OBP in Iran, ultimately enhancing patient outcomes and strengthening the role of OT in rehabilitation.

### 6.1. Limitations

This study, with a major strength, provides a comprehensive evaluation of OBP implementation among various factors including education, workplace, and experience. The large sample size (*N* = 202) also enhances the credibility of the findings and allows for the generalization of the results. However, the cross-sectional design of the study prevents precise causal inference, and the causal relationship between the variables cannot be definitively determined. Additionally, the use of self-reported data can lead to social desirability bias and overstate positive attitudes towards OBP.

## Figures and Tables

**Table 1:** Demographic variables of participants (*n* = 202).

**Variable**	**Category**	**No**	**%**
Gender	Male	80	39.6
	Female	122	60.4

Educational degree	Bachelor	106	52.5
	Master	74	36.6
	PhD	22	10.9

Clinical experience	Less than 5 years	102	50.5
	5–10 years	54	26.7
	10–15 years	20	9.9
	More than 15 years	26	12.9

Field of work	Mental health	110	54.5
	Physical health	145	71.8
	Psychiatry	15	7.4

Place of service provision	Private center	136	67.3
	Government center	22	10.9
	Private and government center	44	21.8

**Table 2:** The mean score of usage rate, environmental factors, therapist's attitude, therapist's knowledge, therapist's interest, and occupation-based practice (OBP) performance among Iranian occupational therapists (*n* = 202).

	**Mean**	**SD**	**Min**	**Max**
Usage rate	26.7	7.14	9	44
Environmental factors	17.9	6.49	2	40
Therapist's attitude	30.1	5.14	14	36
Therapist's knowledge	10.5	3.24	0	16
Therapist's interest	9.4	1.82	5	12
Total performance	94.8	18.1	40	130

**Table 3:** The mean score and percentage of environmental factors influencing occupation-based practice (OBP) performance among Iranian occupational therapists (*n* = 202).

**Environmental factors influencing OBP**	**Mean**	**SD**	**Min**	**Max**	**Scale (%)**
Compatibility of the community's physical environment with OBP implementation	1.3	0.97	0	4	32.5
Motivation from clients and families for implementing OBP	2.3	0.94	0	4	57.5
Motivation from healthcare professionals for implementing OBP	1.9	1.02	0	4	47.5
Administrator cooperation in the implementation of OBP	2	1.05	0	4	50
Implementation of OBP by colleagues in clinical settings	1.8	0.95	1	4	45
Instruction on OBP in practical settings by occupational therapy educators	1.8	1.14	0	4	45
Compatibility of the occupational therapy university curriculum with OBP	2	1.05	0	4	50
Compatibility of clinic space and equipment with OBP implementation	1.6	1	1	4	40
Availability of time for implementing OBP	1.7	0.91	0	4	42.5
Access to occupation-based assessment tools	1.5	1	0	4	37.5
Total environmental factors					44.7

**Table 4:** Mean, SD, minimum, and maximum of total occupation-based practice scores by gender, educational degree, field of work, and place of service provision among Iranian occupational therapists (*n* = 202).

**Variable**	**Category**	**Mean**	**SD**	**Min**	**Max**	**p** ** value**
Gender	Male	93.6	17.67	57	129	0.472
	Female	95.5	18.41	40	130	

Educational degree	Bachelor	91.2	17.3	40	129	< 0.001⁣^∗^
	Master	96.4	17.28	46	126	
	PhD	106.5	19.6	68	130	

Field of work	Mental health	94.7	17.42	40	129	0.49
	Physical health	94.2	17.71	46	130	
	Psychiatry	97.9	14.32	68	113	

Place of service provision	Private center	91.5	18.16	40	129	< 0.001⁣^∗^
	Government center	104.1	18.16	66	126	
	Private and government center	100.1	15.08	65	130	

Total				0	148	

⁣^∗^Statistically significant difference.

## Data Availability

The data that support the findings of this study are available on request from the corresponding author. The data are not publicly available due to privacy or ethical restrictions.

## Endnotes

^1^Occupational Therapy Practice Framework: Domain and Process-Fourth Edition, 2020
